# Chlorido(pentane-2,4-dionato-κ^2^ *O*,*O*′)(1,10-phenanthroline-κ^2^ *N*,*N*′)copper(II)

**DOI:** 10.1107/S1600536809027111

**Published:** 2009-07-18

**Authors:** Yew Sent Wong, Chew Hee Ng, Seik Weng Ng

**Affiliations:** a Faculty of Engineering and Science, Universiti Tunku Abdul Rahman, 53300 Kuala Lumpur, Malaysia; b Department of Chemistry, University of Malaya, 50603 Kuala Lumpur, Malaysia

## Abstract

The Cu^II^ atom in the title compound, [Cu(C_5_H_7_O_2_)Cl(C_12_H_8_N_2_)], shows a distorted square-planar coordination; the chelating N and O atoms occupy the basal sites and the Cl atom the apical site. The square-pyramidal character along the Berry *D*_3h_–*C*_4v_ pseudorotation pathway is 92%.

## Related literature

For the synthesis and electronic spectrum, see: Kwik & Ang (1978 ▶). For isostructural CuBr(C_12_H_8_N_2_)(C_5_H_7_O_2_), see: Onawumi *et al.* (2008 ▶).
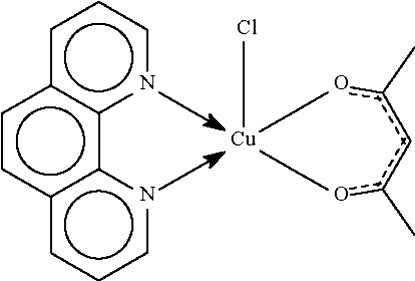


## Experimental

### 

#### Crystal data

[Cu(C_5_H_7_O_2_)Cl(C_12_H_8_N_2_)]
*M* *_r_* = 378.30
Triclinic, 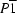

*a* = 7.5436 (1) Å
*b* = 9.0347 (2) Å
*c* = 11.9399 (2) Å
α = 85.638 (1)°
β = 72.329 (1)°
γ = 85.716 (1)°
*V* = 771.97 (2) Å^3^
*Z* = 2
Mo *K*α radiation
μ = 1.60 mm^−1^
*T* = 163 K
0.35 × 0.35 × 0.15 mm

#### Data collection

Bruker SMART APEX diffractometer
Absorption correction: multi-scan (*SADABS*; Sheldrick, 1996 ▶) *T*_min_ = 0.605, *T*_max_ = 0.796
5384 measured reflections
3406 independent reflections
3151 reflections with *I* > 2σ(*I*)
*R*_int_ = 0.015

#### Refinement

*R*[*F* ^2^ > 2σ(*F* ^2^)] = 0.028
*wR*(*F* ^2^) = 0.073
*S* = 1.03
3406 reflections
210 parameters
H-atom parameters constrained
Δρ_max_ = 0.34 e Å^−3^
Δρ_min_ = −0.43 e Å^−3^

### 

Data collection: *APEX2* (Bruker, 2008 ▶); cell refinement: *APEX2*; data reduction: *SAINT* (Bruker, 2008 ▶); program(s) used to solve structure: *SHELXS97* (Sheldrick, 2008 ▶); program(s) used to refine structure: *SHELXL97* (Sheldrick, 2008 ▶); molecular graphics: *X-SEED* (Barbour, 2001 ▶); software used to prepare material for publication: *publCIF* (Westrip, 2009 ▶).

## Supplementary Material

Crystal structure: contains datablocks I, global. DOI: 10.1107/S1600536809027111/xu2552sup1.cif

Structure factors: contains datablocks I. DOI: 10.1107/S1600536809027111/xu2552Isup2.hkl

Additional supplementary materials:  crystallographic information; 3D view; checkCIF report

## Figures and Tables

**Table:** 

Cu1—Cl1	2.4717 (6)
Cu1—O1	1.927 (2)
Cu1—O2	1.936 (2)
Cu1—N1	2.038 (2)
Cu1—N2	2.025 (2)

**Table:** 

O1—Cu1—O2	93.82 (7)
N1—Cu1—N2	81.04 (7)

**Fig. 1.**
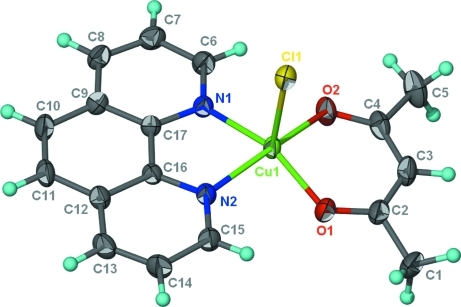
Thermal ellipsoid (Barbour, 2001) plot of CuCl(C12H8N2)(C5H7O2) at the 70% probability level. Hydrogen atoms are drawn as spheres of arbitrary radii.

## supplementary crystallographic information

### Experimental

Copper chloride dihydrate (0.17 g, 1 mmol) and 1,10-phenanthroline monohydrate (0.20 g, 1 mmol) were reacted in a 1:1 *v*/*v* methanol-water mixture (5 ml) to give a light green precipitate. The copper dichloride^.^phenanthroline adduct (0.047 g, 0.15 mmol) was dissolved in a methanol-water mixture (5 ml) and this was treated with excess acetylacetone (5 ml) and 0.1 *M* sodium hydroxide (5 ml). The dark green solution was heated at 343 K for 30 min. Bluish-green crystals separated from the cool solution.

### Refinement

Carbon-bound H-atoms were placed in calculated positions (C—H 0.95–0.98 Å) and were included in the refinement in the riding model approximation, with U_iso_(H) set to 1.2–1.5U_eq_(C).

### Crystal data

**Table:**

[Cu(C_5_H_7_O_2_)Cl(C_12_H_8_N_2_)]	*Z* = 2
*M**_r_* = 378.30	*F*(000) = 386
Triclinic, *P*1	*D*_x_ = 1.627 Mg m^−^^3^
Hall symbol: -P 1	Mo *K*α radiation, λ = 0.71073 Å
*a* = 7.5436 (1) Å	Cell parameters from 4297 reflections
*b* = 9.0347 (2) Å	θ = 2.8–28.3°
*c* = 11.9399 (2) Å	µ = 1.60 mm^−^^1^
α = 85.638 (1)°	*T* = 163 K
β = 72.329 (1)°	Block, blue
γ = 85.716 (1)°	0.35 × 0.35 × 0.15 mm
*V* = 771.97 (2) Å^3^	

### Data collection

**Table:**

Bruker SMART APEX diffractometer	3406 independent reflections
Radiation source: fine-focus sealed tube	3151 reflections with *I* > 2σ(*I*)
graphite	*R*_int_ = 0.015
ω scans	θ_max_ = 27.5°, θ_min_ = 1.8°
Absorption correction: multi-scan (*SADABS*; Sheldrick, 1996)	*h* = −9→9
*T*_min_ = 0.605, *T*_max_ = 0.796	*k* = −11→11
5384 measured reflections	*l* = −15→15

### Refinement

**Table:**

Refinement on *F*^2^	Primary atom site location: structure-invariant direct methods
Least-squares matrix: full	Secondary atom site location: difference Fourier map
*R*[*F*^2^ > 2σ(*F*^2^)] = 0.028	Hydrogen site location: inferred from neighbouring sites
*wR*(*F*^2^) = 0.073	H-atom parameters constrained
*S* = 1.03	*w* = 1/[σ^2^(*F*_o_^2^) + (0.0226*P*)^2^ + 1.0753*P*] where *P* = (*F*_o_^2^ + 2*F*_c_^2^)/3
3406 reflections	(Δ/σ)_max_ = 0.001
210 parameters	Δρ_max_ = 0.34 e Å^−^^3^
0 restraints	Δρ_min_ = −0.43 e Å^−^^3^

### Fractional atomic coordinates and isotropic or equivalent isotropic displacement parameters (Å^2^)

**Table:**

	*x*	*y*	*z*	*U*_iso_*/*U*_eq_	
Cu1	0.74448 (4)	0.62243 (3)	0.25532 (2)	0.01986 (8)	
Cl1	0.42757 (8)	0.74327 (6)	0.30310 (4)	0.02521 (12)	
O1	0.7987 (2)	0.61537 (17)	0.40339 (13)	0.0275 (3)	
O2	0.8805 (2)	0.79922 (17)	0.19426 (14)	0.0272 (3)	
N1	0.7637 (2)	0.56785 (19)	0.08940 (15)	0.0197 (3)	
N2	0.6667 (2)	0.41013 (19)	0.29186 (15)	0.0201 (3)	
C1	0.8971 (4)	0.6884 (3)	0.5587 (2)	0.0391 (6)	
H1A	0.7958	0.6294	0.6087	0.059*	
H1B	0.8954	0.7830	0.5942	0.059*	
H1C	1.0168	0.6335	0.5514	0.059*	
C2	0.8716 (3)	0.7181 (3)	0.4385 (2)	0.0261 (5)	
C3	0.9315 (3)	0.8491 (3)	0.3737 (2)	0.0313 (5)	
H3	0.9691	0.9233	0.4126	0.038*	
C4	0.9407 (3)	0.8800 (2)	0.2562 (2)	0.0256 (5)	
C5	1.0312 (4)	1.0189 (3)	0.1944 (2)	0.0370 (6)	
H5A	1.0362	1.0225	0.1113	0.056*	
H5B	1.1580	1.0185	0.2005	0.056*	
H5C	0.9584	1.1063	0.2314	0.056*	
C6	0.8170 (3)	0.6495 (2)	−0.01113 (19)	0.0243 (4)	
H6	0.8574	0.7464	−0.0101	0.029*	
C7	0.8159 (3)	0.5982 (3)	−0.11824 (19)	0.0270 (5)	
H7	0.8561	0.6597	−0.1884	0.032*	
C8	0.7568 (3)	0.4593 (3)	−0.12245 (18)	0.0247 (4)	
H8	0.7551	0.4239	−0.1950	0.030*	
C9	0.6982 (3)	0.3695 (2)	−0.01631 (18)	0.0213 (4)	
C10	0.6357 (3)	0.2224 (2)	−0.00981 (19)	0.0254 (4)	
H10	0.6279	0.1814	−0.0790	0.030*	
C11	0.5872 (3)	0.1408 (2)	0.0943 (2)	0.0263 (5)	
H11	0.5476	0.0430	0.0966	0.032*	
C12	0.5948 (3)	0.1995 (2)	0.20076 (18)	0.0216 (4)	
C13	0.5500 (3)	0.1196 (2)	0.3117 (2)	0.0266 (5)	
H13	0.5100	0.0210	0.3198	0.032*	
C14	0.5653 (3)	0.1866 (2)	0.40724 (19)	0.0267 (5)	
H14	0.5371	0.1341	0.4820	0.032*	
C15	0.6226 (3)	0.3326 (2)	0.39506 (18)	0.0230 (4)	
H15	0.6302	0.3778	0.4626	0.028*	
C16	0.6529 (3)	0.3440 (2)	0.19648 (17)	0.0183 (4)	
C17	0.7057 (3)	0.4300 (2)	0.08651 (17)	0.0183 (4)	

### Atomic displacement parameters (Å^2^)

**Table:**

	*U*^11^	*U*^22^	*U*^33^	*U*^12^	*U*^13^	*U*^23^
Cu1	0.02849 (15)	0.01578 (13)	0.01782 (13)	−0.00745 (10)	−0.00941 (10)	−0.00008 (9)
Cl1	0.0299 (3)	0.0241 (3)	0.0245 (2)	−0.0016 (2)	−0.0116 (2)	−0.00471 (19)
O1	0.0404 (9)	0.0230 (8)	0.0251 (8)	−0.0066 (7)	−0.0179 (7)	−0.0008 (6)
O2	0.0357 (9)	0.0206 (8)	0.0261 (8)	−0.0129 (7)	−0.0080 (7)	−0.0005 (6)
N1	0.0240 (9)	0.0180 (8)	0.0181 (8)	−0.0041 (7)	−0.0076 (7)	0.0001 (6)
N2	0.0254 (9)	0.0168 (8)	0.0189 (8)	−0.0031 (7)	−0.0075 (7)	−0.0007 (6)
C1	0.0551 (17)	0.0374 (14)	0.0368 (13)	0.0050 (12)	−0.0312 (13)	−0.0112 (11)
C2	0.0282 (11)	0.0258 (11)	0.0291 (11)	0.0042 (9)	−0.0149 (9)	−0.0102 (9)
C3	0.0356 (13)	0.0265 (12)	0.0385 (13)	−0.0064 (10)	−0.0179 (11)	−0.0102 (10)
C4	0.0211 (10)	0.0200 (10)	0.0355 (12)	−0.0043 (8)	−0.0060 (9)	−0.0071 (9)
C5	0.0362 (13)	0.0262 (12)	0.0447 (14)	−0.0134 (10)	−0.0020 (11)	−0.0083 (10)
C6	0.0290 (11)	0.0211 (10)	0.0238 (10)	−0.0082 (8)	−0.0092 (9)	0.0032 (8)
C7	0.0327 (12)	0.0289 (12)	0.0200 (10)	−0.0064 (9)	−0.0093 (9)	0.0051 (8)
C8	0.0282 (11)	0.0293 (12)	0.0183 (9)	−0.0032 (9)	−0.0088 (8)	−0.0022 (8)
C9	0.0209 (10)	0.0231 (10)	0.0210 (10)	−0.0020 (8)	−0.0073 (8)	−0.0018 (8)
C10	0.0308 (11)	0.0237 (11)	0.0246 (10)	−0.0040 (9)	−0.0105 (9)	−0.0073 (8)
C11	0.0335 (12)	0.0188 (10)	0.0291 (11)	−0.0075 (9)	−0.0110 (9)	−0.0046 (8)
C12	0.0237 (10)	0.0187 (10)	0.0221 (10)	−0.0032 (8)	−0.0059 (8)	−0.0016 (8)
C13	0.0327 (12)	0.0179 (10)	0.0280 (11)	−0.0058 (9)	−0.0071 (9)	0.0017 (8)
C14	0.0350 (12)	0.0213 (11)	0.0230 (10)	−0.0057 (9)	−0.0078 (9)	0.0049 (8)
C15	0.0289 (11)	0.0218 (10)	0.0185 (9)	−0.0042 (8)	−0.0070 (8)	0.0004 (8)
C16	0.0190 (9)	0.0163 (9)	0.0196 (9)	−0.0013 (7)	−0.0058 (7)	−0.0011 (7)
C17	0.0184 (9)	0.0177 (9)	0.0195 (9)	−0.0022 (7)	−0.0065 (7)	−0.0010 (7)

### Geometric parameters (Å, °)

**Table:**

Cu1—Cl1	2.4717 (6)	C5—H5C	0.9800
Cu1—O1	1.927 (2)	C6—C7	1.396 (3)
Cu1—O2	1.936 (2)	C6—H6	0.9500
Cu1—N1	2.038 (2)	C7—C8	1.372 (3)
Cu1—N2	2.025 (2)	C7—H7	0.9500
O1—C2	1.271 (3)	C8—C9	1.420 (3)
O2—C4	1.273 (3)	C8—H8	0.9500
N1—C6	1.328 (3)	C9—C17	1.399 (3)
N1—C17	1.357 (3)	C9—C10	1.433 (3)
N2—C15	1.332 (3)	C10—C11	1.359 (3)
N2—C16	1.360 (3)	C10—H10	0.9500
C1—C2	1.507 (3)	C11—C12	1.431 (3)
C1—H1A	0.9800	C11—H11	0.9500
C1—H1B	0.9800	C12—C16	1.401 (3)
C1—H1C	0.9800	C12—C13	1.418 (3)
C2—C3	1.390 (3)	C13—C14	1.370 (3)
C3—C4	1.391 (3)	C13—H13	0.9500
C3—H3	0.9500	C14—C15	1.402 (3)
C4—C5	1.506 (3)	C14—H14	0.9500
C5—H5A	0.9800	C15—H15	0.9500
C5—H5B	0.9800	C16—C17	1.435 (3)
			
O1—Cu1—O2	93.82 (7)	H5B—C5—H5C	109.5
O1—Cu1—N2	88.87 (7)	N1—C6—C7	122.4 (2)
O2—Cu1—N2	164.40 (7)	N1—C6—H6	118.8
O1—Cu1—N1	158.42 (7)	C7—C6—H6	118.8
O2—Cu1—N1	91.13 (7)	C8—C7—C6	120.18 (19)
N1—Cu1—N2	81.04 (7)	C8—C7—H7	119.9
O1—Cu1—Cl1	102.88 (5)	C6—C7—H7	119.9
O2—Cu1—Cl1	97.34 (5)	C7—C8—C9	118.7 (2)
N2—Cu1—Cl1	97.03 (5)	C7—C8—H8	120.6
N1—Cu1—Cl1	97.28 (5)	C9—C8—H8	120.6
C2—O1—Cu1	124.95 (14)	C17—C9—C10	119.20 (19)
C4—O2—Cu1	124.08 (15)	C17—C9—C8	116.96 (19)
C6—N1—C17	118.08 (18)	C10—C9—C8	123.8 (2)
C6—N1—Cu1	129.04 (15)	C11—C10—C9	120.7 (2)
C17—N1—Cu1	112.82 (13)	C11—C10—H10	119.7
C15—N2—C16	117.97 (18)	C9—C10—H10	119.7
C15—N2—Cu1	128.74 (15)	C10—C11—C12	121.3 (2)
C16—N2—Cu1	113.24 (13)	C10—C11—H11	119.4
C2—C1—H1A	109.5	C12—C11—H11	119.4
C2—C1—H1B	109.5	C16—C12—C13	116.90 (19)
H1A—C1—H1B	109.5	C16—C12—C11	118.84 (19)
C2—C1—H1C	109.5	C13—C12—C11	124.2 (2)
H1A—C1—H1C	109.5	C14—C13—C12	119.0 (2)
H1B—C1—H1C	109.5	C14—C13—H13	120.5
O1—C2—C3	125.0 (2)	C12—C13—H13	120.5
O1—C2—C1	115.3 (2)	C13—C14—C15	120.21 (19)
C3—C2—C1	119.7 (2)	C13—C14—H14	119.9
C2—C3—C4	125.1 (2)	C15—C14—H14	119.9
C2—C3—H3	117.4	N2—C15—C14	122.2 (2)
C4—C3—H3	117.4	N2—C15—H15	118.9
O2—C4—C3	125.5 (2)	C14—C15—H15	118.9
O2—C4—C5	116.0 (2)	N2—C16—C12	123.74 (18)
C3—C4—C5	118.4 (2)	N2—C16—C17	116.22 (18)
C4—C5—H5A	109.5	C12—C16—C17	120.03 (19)
C4—C5—H5B	109.5	N1—C17—C9	123.64 (18)
H5A—C5—H5B	109.5	N1—C17—C16	116.42 (18)
C4—C5—H5C	109.5	C9—C17—C16	119.93 (18)
H5A—C5—H5C	109.5		
			
O2—Cu1—O1—C2	10.33 (19)	C6—C7—C8—C9	−0.3 (3)
N2—Cu1—O1—C2	174.95 (19)	C7—C8—C9—C17	−0.2 (3)
N1—Cu1—O1—C2	113.2 (2)	C7—C8—C9—C10	−179.3 (2)
Cl1—Cu1—O1—C2	−88.10 (18)	C17—C9—C10—C11	−1.2 (3)
O1—Cu1—O2—C4	−11.32 (19)	C8—C9—C10—C11	177.9 (2)
N2—Cu1—O2—C4	−110.9 (3)	C9—C10—C11—C12	0.8 (4)
N1—Cu1—O2—C4	−170.31 (18)	C10—C11—C12—C16	0.2 (3)
Cl1—Cu1—O2—C4	92.21 (18)	C10—C11—C12—C13	−178.6 (2)
O1—Cu1—N1—C6	−115.3 (2)	C16—C12—C13—C14	0.1 (3)
O2—Cu1—N1—C6	−12.0 (2)	C11—C12—C13—C14	178.9 (2)
N2—Cu1—N1—C6	−178.4 (2)	C12—C13—C14—C15	0.7 (3)
Cl1—Cu1—N1—C6	85.57 (19)	C16—N2—C15—C14	0.7 (3)
O1—Cu1—N1—C17	67.4 (2)	Cu1—N2—C15—C14	177.97 (16)
O2—Cu1—N1—C17	170.77 (15)	C13—C14—C15—N2	−1.1 (4)
N2—Cu1—N1—C17	4.33 (14)	C15—N2—C16—C12	0.1 (3)
Cl1—Cu1—N1—C17	−91.68 (14)	Cu1—N2—C16—C12	−177.56 (16)
O1—Cu1—N2—C15	17.41 (19)	C15—N2—C16—C17	−178.63 (19)
O2—Cu1—N2—C15	117.6 (3)	Cu1—N2—C16—C17	3.7 (2)
N1—Cu1—N2—C15	178.3 (2)	C13—C12—C16—N2	−0.5 (3)
Cl1—Cu1—N2—C15	−85.43 (19)	C11—C12—C16—N2	−179.4 (2)
O1—Cu1—N2—C16	−165.19 (15)	C13—C12—C16—C17	178.19 (19)
O2—Cu1—N2—C16	−65.0 (3)	C11—C12—C16—C17	−0.7 (3)
N1—Cu1—N2—C16	−4.33 (14)	C6—N1—C17—C9	−0.3 (3)
Cl1—Cu1—N2—C16	91.97 (14)	Cu1—N1—C17—C9	177.29 (16)
Cu1—O1—C2—C3	−3.0 (3)	C6—N1—C17—C16	178.75 (19)
Cu1—O1—C2—C1	178.82 (16)	Cu1—N1—C17—C16	−3.7 (2)
O1—C2—C3—C4	−7.9 (4)	C10—C9—C17—N1	179.7 (2)
C1—C2—C3—C4	170.2 (2)	C8—C9—C17—N1	0.5 (3)
Cu1—O2—C4—C3	5.2 (3)	C10—C9—C17—C16	0.7 (3)
Cu1—O2—C4—C5	−175.75 (16)	C8—C9—C17—C16	−178.49 (19)
C2—C3—C4—O2	6.6 (4)	N2—C16—C17—N1	0.0 (3)
C2—C3—C4—C5	−172.4 (2)	C12—C16—C17—N1	−178.79 (19)
C17—N1—C6—C7	−0.3 (3)	N2—C16—C17—C9	179.10 (19)
Cu1—N1—C6—C7	−177.38 (17)	C12—C16—C17—C9	0.3 (3)
N1—C6—C7—C8	0.6 (4)		

## References

[bb1] Barbour, L. J. (2001). *J. Supramol. Chem.* **1**, 189–191.

[bb2] Bruker (2008). *APEX2* and *SAINT* Bruker AXS Inc., Madison, Wisconsin, USA.

[bb3] Kwik, W. L. & Ang, H. G. (1978). *Aust. J. Chem.* **31**, 459–463.

[bb4] Onawumi, O. O. E., Faboya, O. O. P., Odunola, O. A., Prasad, T. K. & Rajasekharan, M. V. (2008). *Polyhedron*, **27**, 113–117.

[bb5] Sheldrick, G. M. (1996). *SADABS* University of Göttingen, Germany.

[bb6] Sheldrick, G. M. (2008). *Acta Cryst.* A**64**, 112–122. 10.1107/S0108767307043930 18156677

[bb7] Westrip, S. P. (2009). *publCIF* In preparation.

